# Conference Report: COMPASS ’93, EIGHTH ANNUAL CONFERENCE ON COMPUTER ASSURANCE Gaithersburg, MD June 14-17, 1993

**DOI:** 10.6028/jres.098.035

**Published:** 1993

**Authors:** Dolores R. Wallace, Elizabeth B. Lennon

**Affiliations:** Computer Systems Laboratory, National Institute of Standards and Technology, Gaithersburg, MD 20899-0001

## 1. Introduction

Cosponsored by the IEEE Aerospace and Electronics Systems Society and the IEEE National Capital Area Council, in cooperation with the British Computer Society, COMPASS is an organization which advances the theory and practice of building computer assurance into critical systems. NIST’s Computer Systems Laboratory hosted the Eighth Annual Conference on Computer Assurance (COMPASS ’93) on June 14–17, 1993, and served as cosponsors with the following industry and government organizations: Area Systems, Inc.; ARINC Research Corporation; Control Systems Analysis, Inc.; CTA, Inc.; IBM; Logicon, Inc.; Naval Research Laboratory; Naval Surface Warfare Center; Systems Safety Society; TRW Systems Division; and the U.S. General Accounting Office. COMPASS ’93 attracted more than 130 participants from government, industry, academia, and foreign countries such as the United Kingdom, Italy, and Taiwan. This year’s theme, “Practical Paths to Assurance,” highlighted the need to use a pragmatic and realistic approach when providing assurance in a computer system.

## 2. Tutorials

COMPASS ’93 featured tutorials for conference participants who wanted an in-depth discussion of two relevant topics. John Rushby and Patrick Lincoln, SRI International, provided an introduction to formal methods with special focus on the use of automated support tools such as PVS, a Prototype “next generation” Verification System that attempts to provide the benefits of powerful and effective automation for an expressive specification language. Worked examples were demonstrated “live” and included examples from hardware design, fault tolerance, and real-time systems. Janet Cugini, NIST, gave a second tutorial on the draft Federal Criteria for Information Technology Security which covered background, protection profiles. Trusted Computing Base (TCB) functional components, development assurance requirements, evaluation assurance requirements, and future work.

The first full day of the conference opened with welcoming remarks by James Burrows, CSL Director; Judith Bramlage, COMPASS ’93 General Chair; and John Marciniak, COMPASS ’93 Program Chair.

## 3. Myths of Dependable Computing

Peter Neumann, SRI International, keynoted the first day of the conference with a talk entitled “Myths of Dependable Computing: Shooting the Straw Herrings in Midstream.” Citing the belief that most users are doing what they can to assure dependable computing, Neumann identified five problem areas: unsecured PCs; distributed systems which do not back up well; standards and criteria which are useful but inadequate; the lack of use of software engineering and fault tolerance; and the fact that the state-of-the-art in software engineering is not being used in critical systems. Stating that myths tend to be true in the small view, false in the larger picture, he discussed the reliability, security, and safety of software systems, and proposed solutions through research, development, and education. Neumann challenged organizations to do it right in the first place, to make hard decisions, and to look at assurance in the context of the larger picture.

## 4. Verification Technology

Moderated by Connie Heitmeyer, Naval Research Laboratory, this session featured three papers. Sidney Bailin and Scott Henderson, CTA Incorporated, presented a talk on “A Tool for Reasoning About Software Models” which described the Formal Interconnection Analysis Tool (FIAT). Supporting formal reasoning about software systems via their specification diagrams, FIAT decomposes the specification into a diagram of interconnected lower-level components and uses this information to establish some automated verification results.

Chung-Ming Huang, National Cheng Kung University, Tainan, Taiwan, described a protocol verification method which could use ESTELLE and SDL verification tools to assure the correctness of communication protocols. Current verification protocol techniques utilize a global state reduction technique to allow the use of ESTELLE and SDL; the new technique reduces global states to a single state for live variables having the same value, making the incremental protocol model applicable in the ESTELLE and SDL verification tools.

The third presenter was Farn Wang of the University of Texas. Building off the successful use of propositional temporal logic in the verification of digital circuits, Wang and co-author Aloysius K. Mok proposed a technique called Asynchronous Register Temporal Logic (ARTL) to address the verification of distributed real-time software systems. ARTL uses a multi-clock model, utilizes reasoning about items which have more than binary values normally found in digital circuits, and uses a freezing modal operator for fixing register contents. Results are promising for using the verifier for larger benchmarks.

## 5. Special Topics

Peter Neumann, session moderator, introduced Carol Taylor, National Security Agency (NSA), who spoke on “Global Protection against Limited Strikes (Trusted Software Methodology).” In a joint effort with General Electric and AT&T, NSA’s task was to solve the information security problems for the Strategic Defense Initiative (SDI). Taylor stressed that in the process of identifying threats and developing safeguards and countermeasures, 85 percent of the methodology was simply good software engineering practices. She identified three requirements for security: the environment to control separation of duties; the trusted process; and vigilance to ensure the integrity of the environment and the process. Increased costs for up-front efforts were largely recovered in the code and test phase.

John McHugh, University of North Carolina, and Greg Chisholm, Argonne National Laboratory, presented a paper on the “Application of the High Trust Process Model to Complexify Management and System Architecture in the SDI.” Despite the expenditure of large amounts of time, money, and effort, the lack of a definitive, detailed architecture indicates the difficulty of the problem. The authors described a risk-driven approach which utilizes a product model and a process model, concluding that prototyping is the primary risk mitigation process and success is more a matter of accident than design.

“Using Ada in Secure Systems” was the topic of a paper by Roberta Gotfried and David J. Naiditch, Hughes Aircraft Company. Gotfried gave an overview of research into applications of Ada to real-time systems, concluding that Ada has advantages for trusted systems implementations generally lacking in other programming languages.

## 6. The Government Accounting Office (GAO) Perspective

Rona Stillman, Chief Scientist, GAO, gave the second-day keynote address on the task at GAO to ensure that the taxpayer is well served by federal information systems. Are systems performing their intended functions and are they performing well? Problems to be addressed include the poor understanding of software as a product; the difficulty in measuring security; the software development process; and security lapses, both physical and operational. GAO is working on defining methodologies for audits. Assurance will be more difficult in the future with the expansion of the national information infrastructure.

## 7. Reliability Measurement

Moderator Reginald Meeson, Institute for Defense Analysis, introduced Herb Hecht, SoHaR, Inc., who spoke on “Rare Conditions–An Important Cause of Failures.” Hecht’s premise was that rarely executed code has a much higher failure rate than frequently executed code during the early operational period. The inability to handle multiple rare conditions is a prominent cause of program failure in well-tested systems. Problem-solving approaches include correcting identified deficiencies; conducting stress testing in accordance with specified procedures; and conducting testing in a high-workload environment with scenarios that emphasize computer and peripheral equipment failures.

Jeffrey Voas, Jeffrey Payne, and Christoph Michael, Reliable Software Technologies Corporation, and Keith Miller, College of William and Mary, presented a paper entitled “Experimental Evidence of Sensitivity Analysis Predicting Minimum Failure Probabilities.” They presented a theoretical statistical technique complementary to black-box testing known as “sensitivity analysis.” While black-box testing establishes an upper limit on the probability of failure, software sensitivity analysis sets a lower limit on the likelihood of failure. Together, these estimates can establish confidence that software does not contain faults.

“Assigning Probabilities for Assurance in Multi-Level Secure (MLS) Database Design” was the subject addressed by Lucian Russell, Argonne National Laboratory. Russell considered the problem in computer assurance that exists when a MLS application is designed using a MLS database. A system of assigning risks to the release of data collections allows database design to be quantified by a risk factor, resulting in reduced costs. A security policy based upon such a rational approach is more likely to gain acceptance for the system.

## 8. System Safety

Michael Brown, Naval Surface Warfare Center, served as session moderator. He introduced Ron Bell, Health and Safety Executive, United Kingdom, who spoke on “Risk and System Integrity Concepts for Safety-Related Control Systems.” Bell gave an overview of the concepts of risk and safety-integrity in relation to safety-related electrical/electronic/programmable electronic systems. Co-authored by D. Reinert of Germany, the presented paper was an abridged version of Annex A of the emerging International Electrotechnical Commission (IEC) Standard “Functional safety of electrical/electronic/programmable electronic systems.” Bell discussed the standards work on the safe utilization of programmable electronic systems (PES) being done in the U.K., Germany, the European Community, and the United States, the major objective of which is the achievement of international standardization.

“Identifying Generic Safety Requirements” was the topic addressed by Jan Filsinger, Booz-Allen & Hamilton, and Jody Heaney, MITRE Corporation. They proposed a four-step approach to identify generic safety requirements: identification of the safety application domains; analysis of the identified domains; a policy and guidance review; and the review of existing tools, techniques, and methodologies. The approach builds upon lessons learned from the security engineering field to provide tentative answers for several outstanding questions in the safety field.

Keith Gallagher, NIST and Loyola College, and James Lyle, NIST, addressed the issue of “Software Safety and Program Slicing.” They proposed a method that uses program slicing to locate all code that contributes to the value of variables that might be part of a safety critical component. They described how slicing-based techniques can be used to validate functional diversity. The researchers are prototyping this method as part of a NIST research project.

## 9. Management and Developmental Issues

Session moderator Charles Payne, NRL, introduced the first paper by Qi Shi, J. A. McDermid, and J. D. Moffett of the University of York. “Developing Secure Systems in a Modular Way” presented a new technique for the development and verification of secure systems in a modular way. At the heart of the technique is a general approach for coping with the composition of modules. The dependencies among modular security requirements are analyzed and used to locate the modules affected by changes, thus avoiding unnecessary security reevaluation of other modules.

James Freeman, CTA, Inc., spoke on security policy modeling. He stressed the production of a formal security policy model as an important element in the development of a secure system. It is important to produce first a system-specific security policy that explicitly identifies only those portions of the system to be modeled. This separation helps to clarify what needs to be specified and reduces ambiguity.

Stephen Cha, Aerospace Corporation, addressed the “Management Aspect of Software Safety.” Cha contended that more immediate and significant effects on current safety-critical projects can be realized by addressing the management aspects first and then the technical ones. Management policies and decisions are critical to software safety because management has the ultimate control over how and when to spend the limited available resources.

## 10. Developing Standards and Issues

Dolores Wallace, NIST, moderated this session. Robin Bloomfield presented an overview of the many draft standards evolving in the European community. The European community is attempting to replace national standards with international standards but has found the process to be time-consuming. There are various standards organizations ranging from those working generically, such as the ISO IEC SC65A working groups 9 and 10 for Functional Safety of Programmable Electronic Systems, to those who are developing application-specific standards, including the nuclear, avionics, and railway signaling industries. The issues of allowing “best practices” or having strong requirements have not been resolved. Another issue is validating that a standard is effective.

The ISO 9000 Standards for Quality, as described by Taz Daughtrey of Babcock and Wilcox, establish requirements for quality management systems for many industries. In Europe, companies must meet ISO 9000 requirements and be registered by an official Registration Accreditation Board in order to market selected products.

Dr. Raghu Singh, of the Navy SPAWAR Command, discussed the draft of the new Military Standard 498, Software Development and Documentation, which harmonizes the previous standards for both weapons systems and information systems. The document has been reviewed by industry, the Department of Defense and other federal agencies and comments have been reconciled into the document. Dr. Singh plans to have approval of the standard by September 30, 1993.

Michael Brown of the Naval Surface Warfare Center reported on the Military Standard 882C, System Safety Program Requirements, and described how it differs from its predecessor, MIL-STD-882B, in both positive and negative aspects. He concluded by stating that while the new standard is an improvement over its predecessor, it may be difficult to apply to projects involving only software.

## 11. Results of Workshops/Studies

Session moderator H. O. Lubbes, Naval Research Laboratory, introduced Chuck Howell, MITRE, who discussed the MITRE Critical Assurance Workshop of September 1992. The workshop indicated a need for risk reduction, a national repository for research problems, personnel exchange, standards development, and equipping developers and certifiers to deal with the formal method.

Susan Gerhart, National Science Foundation, reviewed the International Survey of the Industrial Applications of Formal Methods conducted by NIST in conjunction with the Naval Research Laboratory and the AECB of Canada. The survey concluded that there are significant applications of formal methods, there is evidence of “best practice,” and advanced tools can be used without excessive costs. The survey cited many limitations, however, including inadequate cost models.

Eugene Troy reported on the Federal Criteria Workshop held at Turf Valley, Maryland, on June 2–3, 1993. The workshop concluded that the replacement of the Orange Book should address distributed systems, networks, encryptions, and PC security. Protection profiles should provide more flexibility and the evaluation and certification process were deemed important issues. There is now a Common Criteria Editorial Board comprised of the European community whose three representatives are from the U.K., France and Germany, and Canada and the United States with one representative each from NIST and NSA. The Board’s objective is to align the three existing IT security criteria documents into a common set of criteria.

## 12. Evening Sessions

COMPASS ’93 also held two social/business activities. On the first evening, two roundtable parallel sessions gave participants the opportunity to discuss issues on Processes (Capability Maturity Model [CMM]) and Standards for Formal Methods. John Baumert, Computer Sciences Corporation, led a discussion on the CMM and explained the differences between the CMM usage and software process assessments where improvement is the principal objective. Roger Fujii, Logicon, proposed different types of formal methods for requirements, design, code and test activities and asked participants to discuss the state-of-the-art of these methods relative to maturity for standardization.

Following the COMPASS banquet the next evening. Dr. Dorothy Denning, Georgetown University, described the Clipper chip, the government-developed “key escrow” chip cryptographic technology, and how it would be used with respect to phone-tapping. Dr. Denning fielded many difficult questions from her audience who were concerned with issues ranging from individual privacy rights to export controls to production of the chip by a single company.

## 13. COMPASS ’94

COMPASS ’94 will be held June 27–30, 1994, at NIST in Gaithersburg, Maryland. The deadline for papers submitted for COMPASS ’94 is January 15,1994. For the first time, the COMPASS organization will give a special award for the best paper that uses technology presented at a previous COMPASS conference; the paper may be research-based or application-oriented. For information about COMPASS ’94 or how to obtain the proceedings of COMPASS ’93, contact Dolores Wallace, Computer Systems Laboratory, National Institute of Standards and Technology, Building 225, Room B266, Gaithersburg, MD 20899-0001; telephone (301) 975-3340 or fax (301) 926-3696.

